# Differentiated Rhabdomyomatous Tumor in a Therapy-Naïve Testicular Germ Cell Tumor

**DOI:** 10.7759/cureus.116606

**Published:** 2026-09-20

**Authors:** Jordan Sarver, Alexander Benben, Timothy Smith, Kenneth Lim, Khaleel I Al-Obaidy

**Affiliations:** 1 Urology, Detroit Medical Center, Detroit, USA; 2 Pathology, McLaren Regional Medical Center, Flint, USA

**Keywords:** orchiectomy, sarcoma, surgery, testicular cancer, urologic oncology

## Abstract

Sarcomatous transformation in testicular germ cell tumors is rare and most commonly associated with post-chemotherapy teratoma. Rhabdomyomatous differentiation is particularly uncommon and typically identified in retroperitoneal lymph node dissection (RPLND) specimens rather than within the primary testicular tumor. We report a unique case of a therapy-naïve 23-year-old male presenting with syncope, dyspnea, abdominal symptoms, and a large right testicular mass. Imaging demonstrated a 12 cm testicular tumor with pulmonary masses suspicious for metastasis. Radical orchiectomy revealed a post-pubertal teratoma containing a predominant (70-80%) differentiated rhabdomyomatous tumor, without mitotic activity, necrosis, or primitive sarcomatous elements. Rare foci of choriocarcinoma were also present. Subsequent jejunal and lung biopsies confirmed metastatic choriocarcinoma. This case represents a rare presentation of a differentiated rhabdomyomatous tumor identified within a primary orchiectomy specimen prior to chemotherapy. Recognition of this rare morphology is clinically relevant, as cytodifferentiated rhabdomyomatous tissue may behave differently from true rhabdomyosarcoma. This report expands the spectrum of testicular germ cell tumor differentiation and contributes to the limited literature regarding sarcomatous evolution in genitourinary malignancy.

## Introduction

Testicular tumors comprise several subtypes. Among these, testicular germ cell tumors can present as either seminomatous or non-seminomatous, with testicular teratoma belonging to the latter group [1]. Sarcomatous components in germ cell tumors are uncommon, with only a few documented cases, and typically result from somatic transformation of a non-seminomatous germ cell tumor, particularly teratomas [1-3]. These sarcomatous components may appear either at initial presentation or following chemotherapy. A testicular rhabdomyosarcoma is a non-seminoma germ cell tumor that arises from a teratoma and develops somatic changes [4].

Rhabdomyosarcoma, although rare, is the most common and aggressive form of sarcomatous transformation and approximately 7% of cases originate in the testes [2]. When this pathology is identified in a retroperitoneal lymph node dissection (RPLND) specimen, it may be associated with poor prognosis, as the presence of sarcoma generally indicates a worse outcome in patients with germ cell tumors [5-7]. In rare cases, mature skeletal muscle is observed on pathology, and previous studies have explored whether these unusual findings behave more like teratomas or true sarcomas, especially when extensive [8]. We herein present a rare testicular tumor in which a mature-appearing rhabdomyomatous component is observed in the primary testicular specimen of a therapy-naïve patient.

## Case presentation

The patient is a 23-year-old male who presented to the emergency room with syncope, shortness of breath, and vomiting. His initial hemoglobin was low, and 2 units of red blood cells (RBCs) were transfused (Table 1). On physical examination, there was a large tender right testicular mass. Tumor markers were obtained and showed an elevated beta-human chorionic gonadotropin (β-hCG), elevated alpha-fetoprotein (AFP), and lactate dehydrogenase (LDH), which were consistent with a possible testicular neoplasm (Table 1). CT angiography of the chest, abdomen, and pelvis revealed “a large right testicular mass measuring at least 12x10 cm, along with multiple lobulated masses in the right lower lung lobe, the largest measuring 4.1 cm. These findings were concerning for a testicular neoplasm with metastatic disease.

**Table 1:** Patient's lab values drawn on initial presentation and follow-up after orchiectomy. AFP: alpha-fetoprotein; β-hCG: beta-human chorionic gonadotropin; LDH: lactate dehydrogenase

Laboratory tests	Patient’s value	Reference range
Initial presentation		
Hemoglobin (Hb)	5.6 g/dL	13.5-17.5 g/dL
Creatinine	0.80 mg/dL	0.7-1.3 mg/dL
White blood cell (WBC)	18.33x10^9^/L	4.0-11.0x10^9^/L
β-hCG	12,296 mIU/mL	<5 mIU/mL
AFP	146 ng/mL	<10-25 ng/mL
LDH	264 U/L	<250 U/L
Follow-up laboratory tests		
β-hCG	118 mIU/mL	<5 mIU/mL
AFP	26 ng/mL	<10-25 ng/mL

The patient then underwent a right radical orchiectomy during the same hospital admission. Microscopically, the tumor showed a non-seminomatous germ cell tumor, formed predominantly of teratoma, post-pubertal type. The teratomatous elements included glandular, cartilaginous, and cystic structures lined by keratinized squamous epithelium, along with a prominent rhabdomyomatous proliferation, occupying approximately 70-80% of the tumor volume. The latter component was composed of nodular and infiltrative aggregates of differentiated rhabdomyocytes, the nature of which was supported by positive immunoreactivity for myogenin immunostain. No evidence of mitotic activity, necrosis, or associated primitive/undifferentiated elements to support a diagnosis of sarcomatous malignancy was identified. Additionally, rare foci of choriocarcinoma were identified, supported by the positive immunoreactivity for GATA3 and β-hCG.

Gastroenterology was consulted due to the patient's anemia on admission, and they performed endoscopic evaluation for concerns of blood loss. The patient underwent an esophagogastroduodenoscopy (EGD) that showed no evidence of bleeding and minimal gastritis. A follow-up colonoscopy with small bowel enteroscopy was completed and showed a large polyp-like mass in the jejunum, which was biopsied and showed clusters of malignant tumor cells with very pleomorphic nuclei, prominent nucleoli, and abundant clear/eosinophilic cytoplasm, with some cells showing multinucleation. By immunohistochemistry, the tumor cells were positive for pan-keratin (clone AE1/AE3), GATA3, β-hCG, focal SALL4, and rare p63, while reactions for OCT3/4, KIT (CD117), CD30, AFP, and glypican 3 were negative. Additionally, the patient underwent a CT-guided lung biopsy, which showed small nests of malignant tumor cells with morphology and immunohistochemical profile similar to those seen in the jejunum biopsy, i.e., choriocarcinoma.

The final pathological staging for this malignancy was testicular germ cell tumor comprised of teratoma, including differentiated rhabdomyomatous tumor and choriocarcinoma, with metastasis to the small intestine and lung, pT2N0M1b (Figures 1A-1D). Following orchiectomy, repeat tumor markers of β-hCG and AFP decreased, consistent with a decrease in overall tumor burden (Table 1). This is reflected in his S1 staging post-orchiectomy. He did undergo chemotherapy with three cycles of cisplatin, etoposide, and bleomycin and is currently completing his fourth cycle, which is three months after his initial diagnosis. His final tumor markers, two months after orchiectomy, normalized. His last CT scan showed no progression or recurrence of disease.

**Figure 1:**
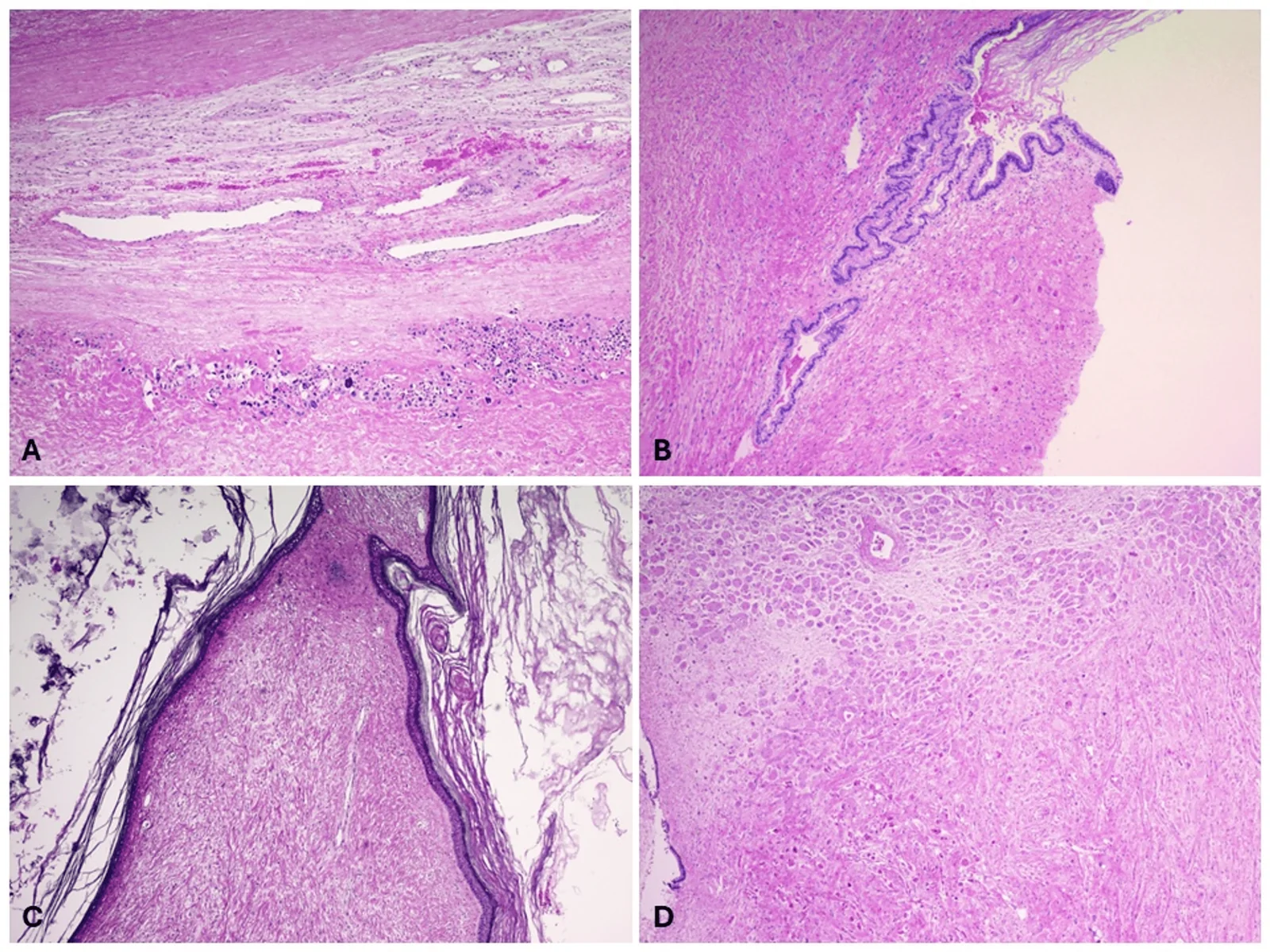
Representative images of the orchiectomy specimen showing foci of residual choriocarcinoma (A), glandular (B), squamous (C), epithelium of the teratomatous component, as well as the background of differentiated rhabdomyomatous cells (D). (H&E 40x).

## Discussion

This study presents a patient with a testicular mass, lung nodules, and a small bowel mass who was diagnosed with metastatic testicular germ cell tumor, including a differentiated rhabdomyomatous component. There are few published reports describing mixed germ cell tumors with a sarcomatous component, and the proposed pathogenesis includes malignant transformation of teratomatous elements, among other mechanisms [9-11]. Non-seminomatous tumors often present with advanced clinical disease, and lymphovascular invasion is a known risk factor for metastatic progression [12]. The presence of sarcomatous elements is particularly significant, as it has been associated with worsened clinical outcomes [13].

Rhabdomyomatous tumors are most commonly identified in post-chemotherapy RPLND specimens, though still rare, accounting for roughly 1% of retroperitoneal lymph node dissections [8]. Prior studies indicate that metastatic germ cell tumors with rhabdomyomatous features behave more clinically like teratoma rather than primary rhabdomyosarcoma [8]. This case is notable in that the patient presented with de novo, therapy-naïve metastatic disease, highlighting the need for continued research into early-stage and atypical presentation patterns.

Clevenger et al. evaluated post-chemotherapy RPLND specimens in seven patients with non-seminomatous germ cell tumors; five contained teratoma, and one demonstrated a focus of embryonal rhabdomyosarcoma [12]. Rhabdomyomatous foci ranged from 0.8 to 5.0 cm [12]. In contrast, the patient in our report underwent upfront radical orchiectomy, and subsequent lung and small bowel biopsies confirmed metastatic germ cell tumor consistent with a testicular primary.

Histologically, rhabdomyomatous tumors are described as nodular or infiltrative aggregates of differentiated rhabdomyocytes, often admixed with teratomatous elements. Cells may appear round with central nuclei or elongated with nuclei positioned peripherally [7]. A component of cytodifferentiation has been proposed in the context of chemotherapy-responsive rhabdomyosarcomatous elements. Prior work comparing pre- and post-chemotherapy specimens demonstrated selective destruction of undifferentiated tumor cells, resulting in more conspicuous rhabdomyoblasts [14]. More favorable outcomes have been observed in cases demonstrating cytodifferentiation [15-17].

Clevenger et al. further proposed that most rhabdomyomatous tumors represent a differentiated element within metastatic germ cell tumor rather than de novo rhabdomyosarcoma [12]. Recognizing the distinction is clinically important, as post-chemotherapy rhabdomyomatous tumors generally lack significant necrosis, brisk mitotic activity, and primitive cellular features characteristic of true rhabdomyosarcoma, and may behave more indolently [16,17].

The presence of sarcoma in RPLND specimens has been associated with worse clinical outcomes. Little Jr et al. reported a disease-specific survival of 68% at 2.5 years [9]. Other authors have described survival rates of ~20% at 1.5 years in patients with sarcomatous transformation in primary or metastatic lesions [6]. Similarly, an overall survival rate of 52% at four years has been reported in teratomas with secondary malignant components, with rhabdomyosarcoma conferring the poorest outcomes [7,18]. Our patient is currently doing well post-operatively. He completed four cycles of chemotherapy and tolerated the therapy well without delay between his treatments. From a urologic perspective, he is recovering appropriately surgically.

## Conclusions

This case reports a very rare and unique presentation of a differentiated rhabdomyomatous tumor identified in a therapy-naïve primary orchiectomy specimen, highlighting both a rare testicular cancer subtype and an unusual pattern of presentation. We hope this case contributes to the limited but growing literature on sarcomatous transformation in germ cell tumors and expands current understanding of rare genitourinary sarcomas.
